# Deep Femoral Artery Branch Pseudoaneurysm After Orthopedic Procedure Requiring Surgical Treatment: A Case Report

**DOI:** 10.5812/traumamon.5181

**Published:** 2012-07-31

**Authors:** Jalalludin Khoshnevis, Mohammad Reza Sobhiyeh, Mahtab Fallah Zavareh

**Affiliations:** 1 Department of General and Vascular Surgery, Shahid Beheshti University of Medical Sciences and Health Services, Shohada Tajrish Hospital, Tehran, IR Iran

**Keywords:** Pseudoaneurysm, False, Deep Artery, Ilizarov Technique

## Abstract

Pseudoaneurysms (PSA) of deep femoral artery (DFA) have been reported following penetrating and blunt trauma to the thigh and orthopedic procedures of the proximal femur. We describe a case of pseudoaneurysm of DFA as a late complication of limb trauma which was confirmed by exploration in an urgent surgery. After two operations successful surgical repair was performed.

## 1. Introduction

Pseudoaneurysms of DFA can present in various forms such as a painful pulsatile mass or even thigh compartment syndrome. As a pulsatile hematoma, pseudoaneurysms, may be the result of any vascular intervention. It may lead to serious consequences, such as major amputation or even death (1). Traumatic pseudoaneurysms of the DFA are only encountered infrequently in sports medical literature and are normally secondary to endovascular interventions or to mycotic infections, in IV drug users or following trauma to the thigh and orthopedic procedures of femur. The majority appear asymptomatically as a pulsatile mass, although on occasions clinical signs of compression (pain, neurological or venous symptoms) may occur; or, if the aneurysm bursts, it can lead to hypovolumic shock (2). Depending on the size of the aneurysm and hemodynamic status of the patient either an urgent or elective repair should be performed (3). We describe a case of femoral fracture with pseudoaneurysm of the DFA that presented with bleeding after orthopedic surgery.

## 2. Case Report

**Figure 1:**
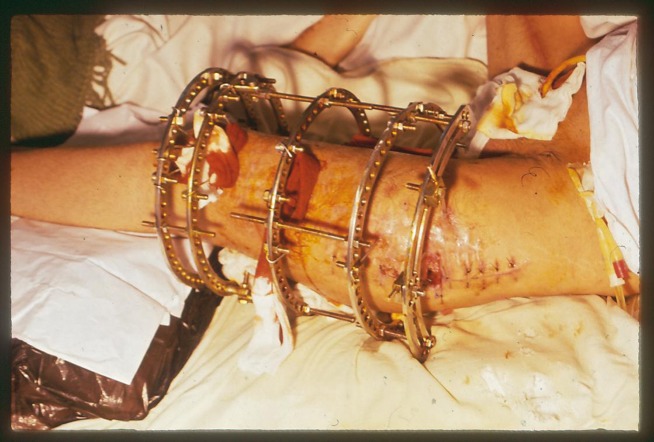
Baround llizarov Apparatus

**Figure 2:**
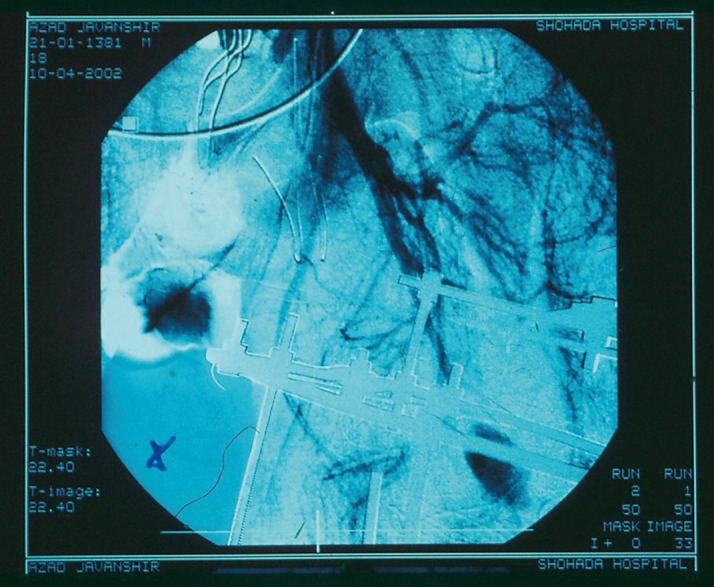
Extravasation of contrast of deep femoral artery branch

**Figure 3:**
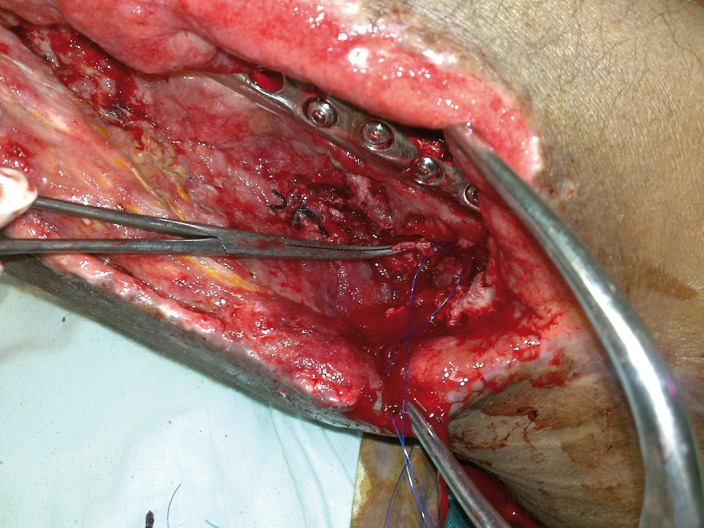
Pseudoanurysm of fhe deep femoral artery

A 25 year-old male motorcyclist had sustained multiple injuries in a road traffic accident including closed fracture of left femoral shaft and lateral condyle and head trauma 1.5 months before recent admission to our hospital. The fracture had been initially treated with an Ilizarov apparatus; however, recently he underwent bleeding around the left lateral femoral surgical incision and was thus referred to our hospital (Figure 1). On arrival, he was hypotensive and had tachycardia. On the examination, his left limb was found atrophic and a 35 cm incision of an orthopedic procedure was lateral to the femur (from proximal toward distal) with granulation tissue and blood oozing out of it with no trill or bruit. Motor examination of fingers and ankle showed impaired dorsiflexion; however the distal pulses in the leg were normal. After stablization of hemodynamic status, exploration of the wound revealed a large hematoma with a bleeding spot from an injured branch of DFA (lateral circumflex femoral artery) and a false aneurysm at that site with communication to DFA. The hematoma was evacuated and the injured branch of deep femoral artery was ligated and bleeding ceased. Ten days after the surgery the patient bled again around his left femur and after performing a contrast-enhanced angiography (Figure 2), in classic exploration (medial approach) of common and deep femoral arteries bleeding from the same branch of DFA was seen. We ligated the injured branch at the point of its communication with DFA where it had originated (Figure 3). The patient had good recovery without further bleeding episodes.

## 3. Discussion

The initial distinction that must be drawn when classifying aneurysms is between “true” and “false” lesions. True aneurysms involve all three layers of the arterial wall, regardless of the underlying pathology. False aneurysms (or “pseudoaneurysms”) are differentiated from true aneurysms by the presence of blood flow outside the normal layers of the arterial wall. At its most basic level, a pseudoaneurysm is simply a hole in an artery that allows extravasation of blood into a contained space outside the artery; it remains pulsatile because of the “to-and-fro” motion of blood in the aneurysm sac. The wall of the false aneurysm is composed of the compressed, surrounding tissues, not the wall of the artery from which the lesion originated (4).PSAs may occur in 4 situations: 1) After catheterization,(2) Synthetic graft anastomoses (e.g., aortofemoral bypass graft),(3) Trauma,(4) Infection (e.g., mycotic PSA). Although the exact incidence is unknown, the risk of spontaneous rupture of PSA is related to the size (3 cm), presence of symptoms, large hematoma, or continued growth of the sac (5). Traumatic pseudo-aneurysms may occur acutely or, may be discovered following unrecognized arterial injury. In all such cases, infection has to be ruled out (6). The diagnostic examination of choice is duplex ultrasound with a 5 - 7 MHz linear transducer. If the puncture site is high (e.g., external iliac artery) or there is extensive hematoma, a lower frequency transducer (curved linear or sector array) may be required to image deeper structures. B-mode scanning alone is unable to differentiate PSA from hematoma. Color Doppler enhances the diagnostic accuracy of Ultrasound by identification of pulsatile flow within the sac (7). Duplex features of femoral pseudoaneurysms include flow outside the artery, the presence of a track or “stalk” from the puncture site to the aneurysm sac, and a characteristic “to-and-fro” flow pattern in the stalk corresponding to blood flow into the aneurysm during systole and sac emptying during diastole (4). The sensitivity of duplex ultrasound to identify a PSA is 94% with a specificity of 97% (8). Management includes direct surgical repair or, in selected instances involving less accessible large arteries, exclusion of the PSA with a stent graft. PSAs arising in small, arteries may be treated with ligation, compression, or coil embolization (6).The close proximity of the DFA to the proximal femur makes it susceptible to damage following fractures and orthopedic procedures. Damage can occur because of bone spikes, drills, screws, displaced implants and even retraction of surrounding tissue. Injury to the profunda DFA accounts for approximately 2% of peripheral arterial wounds. Complications of undiagnosed and inaccessible arterial injuries including PSA, arteriovenous fistula, and vessel occlusion. PSAs may result from blunt, penetrating, or high velocity trauma. Traumatic occlusion of the DFA does not normally result in distal ischemia if the common and superficial femoral arteries are intact. PSAs of the DFA are uncommon and occur as a late complication of various traumas. Causes include iatrogenic (percutaneous or open arterial catheterizations, leakage occurring at anastomoses between grafts and vessels, or orthopedic manipulations), traumatic (blunt, penetrating, and gunshot injuries), and other factors such as infection, hip torsion during sporting activity, intravenous drug usage, and true aneurysms. In our case, the etiology was traumatic. Laceration of an artery can be sealed by a hematoma that may lyse, resulting in formation of a PSA. This is generally characterized by a pulsatile mass connected to the arterial lumen, originating from the surrounding structures as a densely fibrous capsule. Its development may take weeks to months. If untreated, it may rupture at any time or ultimately develop into a chronic aneurysm. DFA injuries may be overlooked due to delayed presentation and also because distal pulses are usually present. Accurate diagnosis is difficult as this artery is located deep in the thigh. The most common clinical presentation of PSA is a pulsating, sometimes painful, mass that expands during systole, usually associated with a strong systolic murmur. Pain and paresthesia, venous occlusion, thrombosis, and edema may develop due to pressure on adjacent nerves and veins (Sidebar 1). Examination and auscultation should be performed over an injured area. DFA injuries may not cause ischemia if the common and superficial femoral arteries are intact. DFA injury is usually only diagnosed arteriographically because overt clinical signs and symptoms are absent. A B-mode ultrasound scan and angiography together with computed tomography or magnetic resonance imaging have been helpful in diagnosis. We initially explored the bleeding site in our patient with no diagnostic procedure because of the patient`s unstable hemodynamic status. If the superficial femoral artery is patent, as was observed in our patient, the PSA could have been treated by simple ligation (9). Indications of surgical repair of femoral false aneurysm are listed in Sidebar 2 (4).Ligation was commonly used in the treatment of vascular injuries during World War II. Ligation of the femoral artery above the DFA resulted in an 81.1% amputation rate compared with 54.8% when the artery was ligated below the PFA. There was no limb loss in our patient. If we had ligated the injured branch of DFA it could have bled. Thus, the correct operation to avoid rebleeding episodes is the ligation of injured branch at its communication with DFA which was the point of its origin in our case. Other therapeutic options include ultrasound-guided compression and trans-catheter embolization (10).

**Sidebar 1:** Indications for Surgical Repair of Femoral Pseudoaneurysms

Infected pseudoaneurysm
Hemodynamic instability
Active bleeding or expanding pulsatile mass
Overlying skin necrosis or cellulitis
Distal limb ischemia
Neurologic deficit (femoral nerve compression)
Failure of ultrasound-guided treatment options
Large aneurysms (> 5 cm) with wide necks

**Sidebar 2:** Factors Associated with the Formation of Pseudoaneurysms

Antiplatelet agents (often aspirin and clopidogrel)
Anticoagulation
Catheterization with large sheath size, 8F
Age 65 years
Obesity
Poor postprocedural compression
Simultaneous artery and vein catheterization
Hypertension
Peripheral arterial disease
Hemodialysis
Complex interventions
Low or high puncture sites
